# Frailty: a global health challenge in need of local action

**DOI:** 10.1136/bmjgh-2024-015173

**Published:** 2024-08-09

**Authors:** Schenelle Dayna Dlima, Alex Hall, Abodunrin Quadri Aminu, Asangaedem Akpan, Chris Todd, Emma R L C Vardy

**Affiliations:** 1 School of Health Sciences, Faculty of Biology, Medicine and Health, The University of Manchester, Manchester, UK; 2 National Institute for Health and Care Research, Applied Research Collaboration - Greater Manchester, School of Health Sciences, Faculty of Biology, Medicine and Health, The University of Manchester, Manchester, UK; 3 National Institute for Health and Care Research Policy Research Unit in Older People and Frailty / Healthy Ageing, School of Health Sciences, Faculty of Biology, Medicine and Health, The University of Manchester, Manchester, UK; 4 Bunbury Regional Hospital, Bunbury, Western Australia, Australia; 5 University of Western Australia, Perth, Western Australia, Australia; 6 Manchester Academic Health Science Centre, Manchester, UK; 7 Manchester University NHS Foundation Trust, Manchester, UK; 8 Oldham Care Organisation, Northern Care Alliance NHS Foundation Trust, Rochdale Road, Oldham, UK

**Keywords:** Global Health, Health policy, Public Health

## Abstract

Frailty is a complex, age-related clinical condition that involves multiple contributing factors and raises the risk of adverse outcomes in older people. Given global population ageing trends, the growing prevalence and incidence of frailty pose significant challenges to health and social care systems in both high-income and lower-income countries. In this review, we highlight the disproportionate representation of research on frailty screening and management from high-income countries, despite how lower-income countries are projected to have a larger share of older people aged ≥60. However, more frailty research has been emerging from lower-income countries in recent years, paving the way for more context-specific guidelines and studies that validate frailty assessment tools and evaluate frailty interventions in the population. We then present further considerations for contextualising frailty in research and practice in lower-income countries. First, the heterogeneous manifestations of frailty call for research that reflects different geographies, populations, health systems, community settings and policy priorities; this can be driven by supportive collaborative systems between high-income and lower-income countries. Second, the global narrative around frailty and ageing needs re-evaluation, given the negative connotations linked with frailty and the introduction of intrinsic capacity by the World Health Organization as a measure of functional reserves throughout the life course. Finally, the social determinants of health as possible risk factors for frailty in lower-income countries and global majority populations, and potential socioeconomic threats of frailty to national economies warrant proactive frailty screening in these populations.

**Summary box**

- Frailty is a multifactorial age-related clinical condition associated with adverse health outcomes, complex health and social care needs, and significant challenges to global healthy ageing goals. However, there is no literature that critiques the current state of frailty research and policy through a global health lens.
- This review bridges the evidence gap by providing a broad overview of frailty in the context of global healthy ageing goals and policies. We highlight how research on frailty screening and management has been predominantly conducted in high-income countries and the lack of geographical and population representativeness, despite rapidly ageing population trends in lower-income countries.
- The evidence base from lower-income countries is showing an increasing trend, and frailty research from these regions should capture the unique contexts, healthcare systems, population dynamics and cultural nuances.
- Further considerations for frailty research in lower-income countries include assessing the narrative around frailty, correlations between frailty and intrinsic capacity in different populations, the social determinants of health as contributing risk factors in global majority populations and the socioeconomic implications of addressing frailty in ageing populations.
- Supportive collaborative networks across high-income and lower-income countries would help drive context-specific research and develop robust care systems for frailty screening and management.

## Introduction

Frailty is a dynamic, age-related clinical condition characterised by losses in physical, physiological, psychological and social functioning.^1^ Frailty impairs an individual’s functional homoeostatic reserve, rendering them more vulnerable to relatively minor acute stressors such as infections and environmental changes.^2^ As frailty is an age-related condition, it poses significant threats to global healthy ageing goals. The world is experiencing a major demographic transition, with the number of older people aged ≥60 projected to double by 2050.^3^ In recognition of the urgency to address population ageing on a global level, increasing attention has been drawn to the concept of healthy ageing, which the World Health Organization (WHO) defined as ‘the process of developing and maintaining the functional ability that enables well-being in older age’,^4^ and that to achieve healthy ageing is to spend the later years of one’s life in good health so that people can meet their basic needs, do what they value and continue contributing to society.^5^ On an individual level, frailty is associated with progressive functional decline and limitations in carrying out basic and instrumental activities of daily living.^6^,^8^ Thus, frailty may impede an individual’s capacity to maintain independence and social engagement, limiting or frustrating their desires to contribute to their communities and do what they value. On a health systems level, frailty is associated with complex healthcare needs as the condition raises the risk of falls, fractures, disability, worsening mobility, loneliness, delirium, dementia and depression, which in turn lead to unplanned hospitalisations, care home admissions, lower quality of life and premature death.^9 10^ This places undue pressure on already strained health and social care systems worldwide.

Frailty affects millions of people worldwide, with global prevalence figures ranging from 12% to 24%, depending on the frailty assessment tools used and the populations included.^11^,^13^ Population ageing transitions are no longer restricted to high-income countries (HICs) in Europe and North America. By 2030, only 20% of older people aged >60 worldwide will live in HICs, and the vast majority will live in lower-income countries^14^ (see box 1 for the glossary of terms used in this review). Given that frailty is an age-related clinical condition, resource-limited health and social care systems in these countries are projected to shoulder a high burden of the complex needs of older people with frailty.

Box 1 **Glossary of terms used in this review**

- High-income countries (HICs): Countries with a gross national income per capita of US$13 846 or higher, according to the World Bank’s income categories.^113^
- Lower-income countries: An umbrella term used in this review to collectively refer to low-income countries, low-income and middle-income countries, and upper-middle-income countries, according to the World Bank’s income categories.^113^
- Context: A spectrum of structural, cultural, natural, political and functional aspects of a social system that influences a person’s social hierarchy and in turn, their health, social and economic opportunities throughout their life.^93 94^
- Deficit accumulation model of frailty: A conceptual model of frailty that proposes ‘the more things individuals have wrong with them, the higher the likelihood that they will be frail’ (Rockwood K, Mitnitski A,^114^, p722). These ‘things that individuals have wrong with them’ are deficits and can include symptoms, signs, disabilities, diseases and laboratory measurements.^114^
- Phenotypic model of frailty: This conceptual model defines frailty as the presence of three or more of the following phenotypes: shrinking (unintentional weight loss of ≥4.5 kg in the past year), weakness (low grip strength), exhaustion (self-reported), slowness (slow walking speed) and low physical activity.^30^
- Social determinants of health: A conceptual framework proposed by the WHO that depicts how social, demographic, political and economic factors influence a person’s health status and well-being, as well as health equity.^93^
- Intrinsic capacity: A composite of a person’s physical and mental capacities (World Health Organization,^75^, p28).
- Functional ability: Comprises a person’s intrinsic capacity, relevant environmental characteristics and the interactions between the person and these characteristics (World Health Organization,^75^, p28).
- Chronic diseases: Also called non-communicable diseases, these tend to affect a person for long durations and can be caused by genetic, physiological, environmental and/or behavioural factors.^115^ Examples include diabetes, cardiovascular diseases, cancer and chronic respiratory diseases.

To date, there is limited evidence that provides a holistic picture of frailty in research, practice and policy in the context of global population ageing goals and strategies. The purpose of our review is to fill this evidence gap by highlighting the current state of frailty in research and clinical practice through a global health and policy lens. By drawing from a large body of literature, we present context-specific implications for addressing frailty as a global health challenge in research, practice and policy.

### Search strategy and evidence synthesis

To understand the geographical representativeness of frailty-related research, we searched PubMed, Google Scholar and Web of Science for different types of reviews that pooled evidence to understand frailty prevalence, incidence, screening, management and guidelines in the last decade. These reviews helped us understand ongoing frailty debates and served as starting points to explore region-specific and country-specific research through citation searches. We excluded reviews that focused on frailty in specific disease populations. We also reviewed reports published by the WHO to understand global healthy ageing goals and strategies. This helped us formulate our arguments for context-specific considerations in the global frailty debate. We primarily used Web of Science and Google Scholar to retrieve frailty-related policy reports and documents for specific countries.

The subsequent sections present an overview of frailty from a global perspective and considerations for contextualising frailty in research, practice and policy.

### Frailty in research and practice from a global perspective

In the frailty discourse, commonly reported challenges are the absence of an internationally recognised frailty definition, lack of standardised frailty assessment tools and interventions, and uncertainties about the true impacts of frailty. These issues are partly attributed to how frailty research is skewed towards specific geographies and economic areas. Most frailty research is from HICs, and the evidence base from lower-income countries is limited and has only been recently growing. Regarding research on the burden of frailty, systematic reviews highlight the disproportionate representation of research from HICs, as exemplified in a systematic review that determined the pooled prevalence of frailty from datasets from 62 countries. Only 2.6% (11/425) of the datasets came from populations in Africa while 36.2% (154/425) datasets came from populations in Europe, followed by the Americas (29.6%), Asia (27.8%) and Oceania (3.8%).^12^ The review also highlighted large in-region and in-country heterogeneities in the population samples. There are wide variations in the frailty research landscape even within continents. For example, in Asia, frailty research is predominantly conducted in Eastern Asia, particularly in Japan and South Korea. Japan is a super-aged society (>20% of the population is aged ≥65)^15^ while South Korea is projected to become one by 2025.^16^ In contrast, other countries in the region have different population structures; for example, 12% of the population in China is aged >65^17^ while the estimate is 5% in Mongolia.^18^ This research trend in Japan and South Korea reflects the national priorities to address the super-ageing populations in these countries. One systematic review estimated the prevalence of frailty and prefrailty in lower-income countries and noted that most studies came from upper-middle-income countries such as Brazil, China, Mexico and Colombia while representation from low-income countries and low-income and middle-income countries was comparatively lower.^13^

Research on frailty management also lacks geographical and population representativeness. Systematic reviews evaluating the effectiveness of frailty interventions have been predominantly conducted in HICs in Europe (such as the UK, the Netherlands, Spain and Sweden) and Asia (such as Japan, South Korea, Hong Kong and Singapore).^19^,^24^ Few trials have been conducted in China, Mexico and Brazil.^19 22 24^ This disproportionately skewed frailty research landscape, compounded by variations in frailty definitions and assessment tools, makes it challenging to establish the true global burden of frailty and to compare the effectiveness of frailty screening and management tools used worldwide.

The characteristics of overall frailty research emerging from lower-income countries and HICs are evidently heterogeneous. Although there are exceptions, frailty research conducted in lower-income countries has predominantly included smaller samples and focused on specific regions and populations within the country to quantify the frailty burden and to adapt different frailty assessment tools. In contrast, frailty research in HICs is exploring large administrative and survey databases to further understand frailty,^25^ determine the prognostic value of frailty for treatment and medical procedures^26 27^ and investigate the biological and genetic characteristics of frailty.^28 29^ Notably, the two predominant conceptualisations of frailty in the literature, the phenotypic and deficit accumulation models, were developed from two population surveys in HICs in North America: the Cardiovascular Health Study in the USA^30^ and the Canadian Study of Health and Aging in Canada.^31^ Population surveys from HICs, such as the Health and Retirement Study in the USA,^32^ English Longitudinal Study of Ageing in England,^33^ Korean Longitudinal Study on Health and Aging,^34^ and Japanese Study of Aging and Retirement,^35^ have been collecting information from as far back as the 1980s, making them data-rich sources for large-scale cross-sectional and longitudinal studies on frailty. In lower-income countries, frailty-specific research has recently shown an increasing trend, aided by the roll-out of population surveys on ageing. Examples include the Health and Aging in Africa: A Longitudinal Study of an INDEPTH Community in South Africa^36^ (baseline survey conducted in 2014–2015), Longitudinal Ageing Study in India (baseline survey conducted in 2017–2018)^37^ and Brazilian Longitudinal Study of Aging (ELSI-Brazil) (baseline survey conducted in 2015–2016).^38^ These surveys enable cross-country comparisons while ensuring the capturing of context-specific data. As these surveys mature, we can expect a more enriched frailty research landscape from lower-income countries that leverages more data points, enables generalisability and sheds light on context-specific nuances in frailty prevalence, screening and management.

Since the two seminal frailty conceptualisation publications in 2001,^30 31^ a plethora of frailty assessment tools has emerged (table 1), although developed and validated in populations in high-income contexts. More recently, evidence is emerging on adapting and validating existing frailty screening tools, such as the Edmonton Frailty Scale,^39^ cumulative deficit frailty index^40^ and Clinical Frailty Scale,^41^ in different populations. Efforts are also being directed towards understanding ageing on a global scale. The WHO Study on Global AGEing and Adult Health is an ongoing programme collecting longitudinal data on ageing and health from the following countries: China, Ghana, India, Mexico, the Russian Federation and South Africa.^42^ This data harmonisation exercise will further promote cross-country comparisons of ageing-related metrics, with more frailty-specific peer-reviewed publications expected in the future.

**Table 1:** Characteristics of frailty screening tools commonly cited in the literature

Tool	Country of origin	Components	Cut-off for frailty	Validation population
Clinical Frailty Scale	Canada	Visual chart with 9 graded pictures.	Score of ≥5 out of 9	Older patients who participated in the second stage of the Canadian Study of Health and Aging.^116^
Edmonton Frailty Scale	Canada	9 items capturing cognition, health, hospitalisation, social support, nutrition, mood, function and continence.	Score of ≥8 out of 17	Referral population for comprehensive geriatric assessment in acute care wards, rehabilitation units, day hospitals and outpatient clinics in Edmonton, Alberta, a major Canadian metropolitan centre.^117^
FRAIL Scale	USA	5 items: fatigue; resistance (ability to climb up one flight of stairs); ambulation (ability to walk one block); illness (>5 comorbidities); weight loss (>5%).	Score of ≥3 out of 5	First validated in the African American Health Project participants, a population-based panel study of African Americansfrom two socioeconomically diverse areas of St. Louis, USA.^118^
Frailty Index from Comprehensive Geriatric Assessment	Canada	Frailty index comprises assessments in 10 standard domains.	Mild frailty: 0–7Moderate frailty: 7–13Severe frailty: >13	Rural community-dwelling older people with frailty in the Mobile Geriatric AssessmentTeam trial around Halifax, Nova Scotia, Canada.^119^
Frailty Index of Accumulated Deficits	Canada	Items include symptoms, signs, abnormal laboratory values, disease classifications and disabilities.	Not defined in the first study.	The Canadian Study of Health and Aging, which aimed to understand dementia in older people in Canada.^31^
Fried Scale	USA	5 items: weight loss; slow walking speed; low grip strength; exhaustion; low physical activity.	Score of ≥3 out of 5	The Cardiovascular Health Study, a prospective, observational study of people aged ≥65 in the USA.^30^
Gérontopôle Frailty Screening Tool	France	Composed of an initial questionnaire on signs/symptoms indicating frailty and a second section where the general practitioner expresses their own view about the frailty status of the individual.	Clinical judgement on whether the individual should be classified as frail.	Patients referred to the Gérontopôle Frailty Clinic, Toulouse, France.^120^
Kihon Checklist	Japan	25 items capturing physical strength, nutrition, eating, socialisation, memory, mood and lifestyle. Final score is calculated as a proportion.	Score of ≥0.25 out of 1	Older regular outpatients at the Hospital of the National Center for Geriatrics and Gerontology to follow up chronic conditions in Obu, Japan.^121^
Multidimensional Prognostic Index	Italy	Developed from CGA data by aggregating the total score of the 8 CGA domains.	Low risk: 0.0–0.33Moderate risk: 0.34–0.66Severe risk: 0.67–1.0	Older hospitalised patients in the Geriatric Unit of the Casa Sollievo della Sofferenza Hospital (IRCCS, San Giovanni Rotondo, Italy).^122^
SHARE Frailty Instrument	12 European countries	Items of the Fried scale. The aim was to develop and validate an instrument freely available via web calculators.	Score of ≥3 out of 5	Participants in wave 1 of the SHARE, a population-based survey conducted in 12 European countries.^123^
Tilburg Frailty Indicator	The Netherlands	10 questions on determinants of frailty and diseases, and 15 questions on components of frailty in three domains (physical, psychological and social frailty).	Score of ≥5 out of 15	Community-dwelling individuals aged ≥75 years in Roosendaal, the Netherlands.^124^

ADL, activities of daily living; CGA, comprehensive geriatric assessmentSHARESurvey of Health, Ageing and Retirement in Europe

In clinical and social care practice, frailty screening and management have been the focus only in HICs owing to the large ageing populations and associated healthcare needs, driving their prioritisation in public health and policy. A recent systematic review retrieved clinical guidelines on frailty prevention and management and found that most originated from the UK, the USA and Canada.^43^ The remaining two guideline documents originated from the Asia-Pacific region^44^ and the International Conference on Frailty and Sarcopenia Research.^45^ Regarding real-world practice, the National Health Service in the UK became the first health system globally to integrate mandatory frailty assessment in routine primary care practice using the electronic frailty index, which automatically calculates frailty scores based on diagnostic codes in a patient’s electronic health record.^46 47^ However, variations persist in its uptake and utilisation in practice, with uncertainties in the tool’s accuracy and clinical utility.^48 49^ In Japan, the Long-Term Care Insurance system is a form of mandatory social insurance system that provides preventive and supportive care services to frail and prefrail individuals.^50^ Eligibility is decided by evaluating frailty status using the Kihon Checklist, followed by a multistep process to determine the dependency levels and care needs of potential beneficiaries.^50 51^ Evidence on frailty in clinical practice in lower-income countries is sparse; hence, how frailty is assessed, recorded and addressed by practitioners in these contexts remains unclear. As the manifestations of frailty overlap with those of chronic diseases and disability, identifying and diagnosing frailty as a distinct clinical condition may be limited due to overburdened health workforces and lack of appropriate training, equipment and frailty tools validated in local populations.^52^ For example, Nigeria reportedly has less than 100 geriatricians for 4 million older people.^53^ Therefore, the clinical opportunities to address frailty once it develops are limited in lower-income countries.

### Considerations for contextualising frailty in research and practice

#### ‘Think globally, research locally’

The extensive research on frailty to date is a welcome sign of its recognition as a public and global health concern. The current direction of frailty research in lower-income countries also signals increasing prioritisation in clinical practice and policy. Table 2 presents examples of frailty research in lower-income countries, and how frailty and ageing strategies have been integrated into practice and policy, reflecting these countries’ different national priorities. The evolution in frailty research should not be considered a linear journey, and the frailty research landscape in lower-income countries does not need to ‘catch up’ to that in HICs. The manifestations of frailty in older people are heterogeneous and influenced by different socioeconomic, demographic and clinical factors.^54^ Such a complex, multidimensional condition warrants research that represents different geographies, populations, health systems and community settings. Thus, research conducted in lower-income countries should expectedly have different objectives, study designs and study sample characteristics reflecting the different research and policy priorities, which may be markedly different from those in HICs. Population ageing surveys in lower-income countries can facilitate the development of tailored frailty assessment and management tools that are validated in specific contexts, as they will collect data and evaluate outcomes most pertinent to national health and policy priorities. Existing frailty screening tools and clinical guidelines can serve as blueprints for context-specific validation studies and trials.^55^,^58^ However, frailty screening tools and guidelines originating from HICs also vary in feasibility and quality^4359^,^61^; thus, global evidence should help guide rather than dictate local implementation research and practice. For example, the GeKo–Integrated Service Delivery model for frailty management in Malaysia^62^ is based on the Canadian Preferred Reporting Items for Systematic Reviews and Meta-Analyses model of care.^63^

**Table 2:** Examples of frailty and ageing in research, practice and policy in lower-income countries

Country	Frailty in research	Frailty and ageing in practice and policy
Nigeria	Predictors of frailty and association of frailty with mortality in hospital settings.^125 126^	The Nigerian government set up the National Senior Citizens Centre (NSCC) in 2021 to improve the standards of care for older people in collaboration with other government agencies.^127^The NSCC’s priority areas include ensuring income security, promoting well-being and health, enhancing social inclusion and enabling access to age-friendly services, supportive environments and discounted services.The NSCC already closely collaborates with the United Nations Department of Social and Economic Affairs, which supported a national workshop in Nigeria to develop minimum standards for care homes and carers of older people.
India	Frailty and its correlations with sociodemographic and economic factors.^128 129^Frailty in specific geographical regions in India.^130 131^Frailty in specific disease and/or treatment populations in India.^132^	Under the ‘National Programme for Health Care of the Elderly 2010–2011’, National Centres of Ageing provide high-level tertiary care to older people and include ‘frail elderly clinics’.^133^
China	A review by Ma *et al* provided an overview of frailty research in China.^134^ The research landscape in China has rapidly grown and diversified in the last few years, which includes studies on validating frailty assessments in Chinese populations, understanding frailty epidemiology using different population databases and conducting trials on frailty interventions.	The ‘Healthy China Initiative 2019–2030’ was launched in 2019 to promote a proactive approach to disease prevention, improve health literacy, encourage healthy lifestyles and enhance infrastructures.^135^ One of the major actions is ‘Elderly health promotion campaigns’.
Mexico	Frailty and its associations with different health-related and lifestyle factors.^136 137^Frailty trajectories over a long follow-up period.^138 139^Intrinsic capacity scales and their associations with frailty.^140^	The ‘Pension for the Well-being of Older People’ programme was established in 2019 to extend unremarkable pensions (a form of social protection) to older Mexicans and indigenous populations.^141^
Brazil	Adapting different frailty tools for the Brazilian population.^142 143^Frailty from a health provider perspective.^144^Frailty in specific disease populations such as people living with HIV^145^ and acute low back pain.^146^A scoping review protocol on how frailty is identified and managed in practice in Brazil has also been published recently.^147^	The Brazilian Consensus on Frailty in Older People task force was established in 2005, comprising experts in frailty-related teaching, care, research and management activities.^148^ Objectives of this task force include establishing a national consensus on frailty indicators and conceptual definitions and operationalising frailty to guide teaching, care and research.
Malaysia	Prevalence of frailty and its risk factors in Malaysians.^149^,^151^Validating and comparing different frailty assessment tools in the Malaysian population.^152 153^	GeKo Integrated Service Delivery clinics, first established in 2019, facilitate frailty risk identification and management in primary care.^62^ These clinics involve multidisciplinary teams that provide coordinated services and personalised care plans to older people with frailty in the community. There are 32 clinics in the state of Sarawak as of 2023.

Note: Frailty research studies were searched i on Web of Science using the terms (frail* AND “country name”) in the title and/or abstract.

These countries were purposefully selected to ensure geographic representativeness.

Several studies and reviews advocate for a ‘standardised frailty definition’ and ‘standardised assessment tool and management plan’. An internationally recognised frailty definition is warranted to ensure consistency across research outputs, policy design and clinical practice guidelines. However, frailty assessment tools should capture the multidimensional contributing factors, health system dynamics and feasibility of routine implementation in specific contexts, and these context-specific tools should align with a standardised frailty definition.

### Evaluating the global narrative around frailty and ageing

There is a call to shift the rhetoric of ageing from a unidirectional path to inevitable morbidity to a positive transition where older people can still thrive, contribute to society and maintain meaningful interactions with their community. Regression in frailty states has been reported,^64^,^67^ but whether frailty progresses or regresses depends on various sociodemographic and clinical factors.^7 68 69^ In efforts to establish shared goals for public health response to population ageing, the WHO advocate moving away from a disease-focused model of ageing, including going beyond the traditional view on frailty.^70 71^ Frailty remains associated with negative connotations, being perceived as identity loss, deterioration of status, a synonym for weakness and dependency, and an inevitable part of the ageing process.^72^,^74^ The WHO is drawing focus on the intrinsic capacity of an individual, which in turn determines their functional ability.^75^ Intrinsic capacity is described as the loss in the functioning of multiple health domains (cognition, locomotion, sensorium, psychology and vitality),^76^ overlapping with the clinical characteristics of frailty. Belloni and Cesari pointed out how intrinsic capacity and frailty can be considered as ‘two sides of the same coin’ (Belloni G, Cesari M, p356).^77^ Intrinsic capacity encompasses a person’s physical and mental reserves throughout their life while frailty correlates with greater vulnerabilities to adverse outcomes from losses in multisystemic physiological reserves in later life.^78^ Thus, intrinsic capacity is a measure of what a person has (reserves) rather than what they lack (deficits),^79^ lending the construct a more positive connotation. Intrinsic capacity may also be beneficial in understanding longitudinal changes in residual capacities throughout the life course, especially as most frailty instruments are only validated in older populations.^80^ Research on operationalising intrinsic capacity^81 82^ and the contributing factors,^83 84^ associations with adverse outcomes^85 86^ and role in evaluating interventions^81 87^ is emerging from both HICs and lower-income countries. However, inconsistencies in the measurement models and operationalisation methods prevail,^88^ impeding translations from theory to real-world practice.^77^ One may argue that the shift from frailty to intrinsic capacity may diminish the wide-ranging impacts of frailty on individuals, communities and health systems. Vernon described how the narrative of frailty as a long-term, clinical, identifiable and potentially reversible condition fueled policy implementation in the UK.^89^ Some frailty tools, such as the frailty index, also capture characteristics not included in the intrinsic capacity composite score, such as comorbidities and social vulnerability. Frailty is challenging to distinguish from manifestations of multiple chronic conditions and disability; hence, screening for frailty specifically using validated tools is important to ensure people receive timely intervention. It should be acknowledged that the construct of intrinsic capacity was developed from the need to provide personalised and integrated care plans, and this has also been recognised for frailty. As research on intrinsic capacity is still in its infancy, there are opportunities to develop a standardised and feasible measurement tool. A composite score that integrates both intrinsic capacity and frailty components may be considered. Although such a standardised tool may have global research and policy benefits by facilitating generalisability and consistency, we recommend that this tool rather serves as a blueprint for more context-specific adaptations that would account for national policies, population health priorities and feasibility in community settings. Recent studies have reported associations between intrinsic capacity and different dimensions of frailty.^90^,^92^ Further research on the potential of embedding intrinsic capacity measurement into current frailty screening and chronic disease management pathways, and public perceptions of intrinsic capacity as an ageing-related concept is warranted.

### Considering the social determinants of health in the frailty discourse

Frailty involves a complex interplay of non-modifiable and modifiable risk factors, which may contribute to differing incidence rates, manifestations and trajectories. The WHO’s social determinants of health^93^ have gained a strong foothold in the global health landscape. Although there is a non-modifiable, age-related component in frailty development,^2^ addressing the social determinants of health can help lower the risk of frailty onset and worsening across populations and contexts, especially given how frailty is preventable and reversible. Drawing from this conceptual framework, Sadana *et al*^94^ proposed a preliminary model for healthy ageing and health equity in a global context, consisting of four components: (1) the physical–socio–economic–political or overall context; (2) genetic inheritance and socioeconomic position; (3) intermediary determinants (such as health and social care systems, intrinsic capacity and the built environment) and (4) the outcome of interest (healthy ageing).^94^ Evidence already shows associations between elements of these components (such as the built environment, sociodemographic characteristics and comorbidities) and frailty.^54 95^ Hence, this model for healthy ageing can also be applied to frailty. Given the limited availability of specialised geriatric workforces to identify and treat frailty in lower-income countries, the model’s components could guide research, policy and actionable strategies to achieve the outcome of interest, that is, frailty prevention and regression.

For example, the social determinants of health and the healthy ageing model can be used to address chronic diseases, which are risk factors for frailty and occur at younger ages in lower-income countries and in global majority populations in HICs.^96 97^ Chronic diseases are related to the intermediary determinants of the healthy ageing model. Living with multiple chronic diseases (multimorbidity) is a growing challenge in lower-income countries that demands context-specific guidelines for its diagnosis and management.^98^ Thus, given the correlation between chronic diseases and frailty, frailty incidence rates may be greater in these populations and geographies, and frailty trajectories may begin earlier and progress more rapidly. Integrating opportunistic frailty screening and management into existing models of chronic disease care may be feasible and cost-effective in such contexts, and determinants such as the built environment, lifestyle behaviours and exposures can be considered while designing and implementing interventions for both chronic diseases and frailty. This demonstrates how intrinsic and extrinsic determinants at multiple levels, such as coverage of services to manage chronic diseases, chronological age, ethnicity, comorbidities and lifestyle, are worth considering for frailty prevention and management in health and social care practice.

### Potential socioeconomic benefits of addressing frailty

Whether the benefits of integrating frailty assessment in routine care outweigh the costs to already strained, resource-limited health and social care systems requires consideration. The frailty research landscape is saturated with studies on new assessment tools rather than management strategies.^99^ Thus, actions necessary following frailty identification may still be unclear to healthcare practitioners in lower-income countries, especially given the lack of evidence on frailty management in these contexts. We argue that frailty should still be considered in healthcare practice and policy in lower-income countries for several reasons. First, evidence shows that the onset of frailty is associated with greater healthcare use and subsequent healthcare costs,^100^,^102^ given the multidisciplinary care needs for the condition.^103^ Second, although evidence is mixed, frailty has been shown to correlate with socioeconomic factors such as education levels, income levels, residence status (rural/urban), neighbourhood deprivation levels and ethnicity.^104^,^109^ Thus, large disparities in socioeconomic status in lower-income countries may drive the disproportionate incidence and progression of frailty, further widening health and social inequities. Third, frailty poses threats to the economic productivity of older people aged ≥55, who make up a larger share of the labour workforce globally.^110^ If there is a risk of earlier frailty onset in populations in lower-income countries, prime economically productive years may be spent being frail, with serious implications on public health expenditure and pension contributions. This can have adverse consequences on the long-term economic health of a country. Evidence suggests that an increasing ageing population could drive long-term economic growth in lower-income countries due to accumulation of capital and assets, consumption patterns and contributions to household incomes if they continue to work.^111^ Finally, frailty is a dynamic condition, and research has shown that the likelihood of improving from prefrailty to robust is greater than improving from frailty to robust.^65^,^67^ Identifying early stages of frailty may facilitate timely intervention, thus optimising scope for frailty reversal.^112^ This is also important in hospital settings, as deconditioning associated with hospitalisation and prolonged bedrest are linked with frailty onset and progression. Frailty should be considered in postdischarge pathways to prevent long-term consequences on individuals and their carers; loss of work productivity and subsequent strains on secondary care, community care and economic resources. Thus, national policies and guidelines that support frailty screening and interventions are in the best interests of healthcare systems and labour markets. These policies and guidelines should account for the heterogeneous risk factors and population dynamics, feasibility of frailty screening in specific healthcare settings and cultural considerations for frailty interventions related to exercise and nutrition. Preventing and optimally managing frailty can help healthcare systems steer away from catastrophic costs and ensure older people remain active contributors to national economies and their communities.

### Strengths and limitations of this review

Owing to the large body of literature, our review is limited by its lack of a systematic approach to extract relevant literature and in-depth analyses. However, we drew from evidence from multiple countries and contexts to provide a narrative overview of the breadth of the frailty literature through a global health lens. Our review is also coauthored by clinical and research experts with broad knowledge of frailty and healthy ageing in research, practice and policy, thus providing balanced arguments on frailty and healthy ageing debates.

## Conclusion

Rapid population ageing in lower-income countries poses significant challenges to healthcare systems and national economies. Ensuring that older people spend their later years in good health is crucial to achieve global healthy ageing goals, which include preventing frailty, a condition associated with problematic ageing. Studies on frailty have predominantly originated from HICs, but their findings cannot be simply generalised to lower-income countries owing to the multidimensional risk factors and heterogeneous manifestations of the condition. The growing and diversifying evidence landscape on frailty from lower-income countries in recent years signals the recognition of frailty as a serious public health challenge. However, research should be context-specific and informed by the cultural nuances, underlying social determinants of health, population dynamics, health system structures and policy priorities. Future directions for research, practice, policy in both global and resource-limited contexts include (1) reaching a consensus on an internationally recognised frailty definition; (2) adapting existing frailty screening tools, interventions and clinical guidelines for local populations; (3) exploring intrinsic capacity as an indicator of ageing over the life course, and its associations with frailty and utility in routine health and social care; (4) addressing the social determinants of health at different care levels to prevent frailty onset and worsening in HICs and lower-income countries; (5) evaluating the socioeconomic benefits of addressing frailty on an individual, community, national and global level, thereby helping drive policy change and (6) building supportive collaborative systems between HICs and lower-income countries to develop and improve care systems and drive research with and for older people to prevent frailty onset and progression.

## Data Availability

All data relevant to the study are included in the article.
